# PDK-1 regulates lactate production in hypoxia and is associated with poor prognosis in head and neck squamous cancer

**DOI:** 10.1038/sj.bjc.6604356

**Published:** 2008-06-10

**Authors:** S M Wigfield, S C Winter, A Giatromanolaki, J Taylor, M L Koukourakis, A L Harris

**Affiliations:** 1 Cancer Research UK, Growth Factor Group, Weatherall Institute of Molecular Medicine, John Radcliffe Hospital, University of Oxford, Headington, Oxford OX3 9DU, UK; 2 Departments of Radiotherapy/Oncology and Pathology, Democritus University of Thrace, Alexandroupolis 68100, Greece; 3 Wellcome Trust Centre for Human Genetics, University of Oxford, Roosevelt Drive, Headington, Oxford OX3 7BN, UK

**Keywords:** pyruvate dehydrogenase kinase 1 (PDK-1), hypoxia, hypoxia-inducible factor-1 (HIF-1), pyruvate, lactate

## Abstract

Here we describe the expression and function of a HIF-1-regulated protein pyruvate dehydrogenase kinase-1 (PDK-1) in head and neck squamous cancer (HNSCC). Using RNAi to downregulate hypoxia-inducible PDK-1, we found that lactate and pyruvate excretion after 16–48 h of hypoxia was suppressed to normoxic levels. This indicates that PDK-1 plays an important role in maintaining glycolysis. Knockdown had no effect on proliferation or survival under hypoxia. The immunohistochemical expression of PDK-1 was assessed in 140 cases of HNSCC. PDK-1 expression was not expressed in normal tissues but was upregulated in HNSCC and found to be predominantly cytoplasmic with occasional strong focal nuclear expression. It was strongly related to poor outcome (*P*=0.005 split by median). These results indicate that HIF regulation of PDK-1 has a key role in maintaining lactate production in human cancer and that the investigation of PDK-1 inhibitors should be investigated for antitumour effects.

The upregulation of glycolysis is a well-recognised phenotype of cancer and it is regulated by hypoxia-inducible factor (HIF) in hypoxic conditions. Hypoxia-inducible factor is a heterodimeric transcription factor, consisting of α and β subunits, which directs a broad range of responses in hypoxic cells (Maxwell *et al*, 1997). Both proteins are members of the basic helix–loop–helix superfamily of transcription factors (Wang *et al*, 1995). In the presence of oxygen, two prolyl sites within a central domain of HIF-1α are hydroxylated by prolyl hydroxylase enzymes, which leads to HIF-1α degradation through the von Hippel-Lindau E3 ubiquitin ligase complex and the 26S proteasome (Maxwell *et al*, 2001). Upon activation, the HIF-1 complex binds to target genes at sites containing the core recognition sequence 5′-RCGTG-3′, also known as the hypoxia regulatory element (Minchenko *et al*, 1994), which leads to the upregulation of genes involved in angiogenesis (VEGF), glucose transport (GLUT-1), glycolytic enzymes (Hexokinase 2) and pH regulation (CA9).

Under hypoxic conditions, anaerobic glycolysis is maintained by conversion of pyruvate to lactate, a reaction catalysed by lactate dehydrogenase A (Koukourakis *et al*, 2005a). In cancer cells, pyruvate is transformed into lactate regardless of the presence of oxygen, a process called the Warburg effect (Warburg, 1956). This results in an increased glycolytic rate and shift to lactate production in cancer cells.

A key branch point in the glycolytic pathway is the production of pyruvate, which in anaerobic conditions is metabolised to lactate and in normoxia by pyruvate dehydrogenase (PDH) to acetyl-CoA, the first step in the TCA cycle (Yeaman, 1989; Reed, 2001; Harris *et al*, 2002; Fries *et al*, 2003; Sugden and Holness, 2003). PDH activity is controlled by two regulatory enzymes; pyruvate dehydrogenase kinase (PDK), which phosphorylates and inactivates the enzyme and pyruvate dehydrogenase phosphatase, which dephosphorylates the enzyme to the active form (Kristo *et al*, 2004; Martin *et al*, 2005). Four PDK isoenzymes have been identified in humans and they exhibit tissue-specific expression (Popov *et al*, 1997; Bowker-Kinley and Popov, 1999). Pyruvate dehydrogenase kinase-1 has been detected in the heart (Wu *et al*, 1998; Bowker-Kinley and Popov, 1999; Sugden *et al*, 2000), the pancreatic islets (Sugden *et al*, 2001), the liver, and the skeletal muscle (Peters *et al*, 2001). Conversion of pyruvate to acetyl CoA by PDH is vital in providing a link between glycolysis and the Krebs cycle, and aerobic respiration, and as a donor for fatty acid, ketone body and cholesterol synthesis.

As the human squamous head and neck cancer (HNSCC) is among the most hypoxic of tumours and because there is evidence of substantial differences between cell lines in the hypoxia transcriptome (Kelly *et al*, 2003; Chi *et al*, 2006), we investigated head and neck cancer cell lines to evaluate if there were any genes not previously reported to be hypoxia inducible, aiming then to evaluate function and clinical significance.

Recently, two reports have identified PDK-1 as a hypoxia-responsive protein that regulates the function of the mitochondria under hypoxic conditions by reducing pyruvate conversion to acetyl CoA, resulting in a drop in mitochondrial oxygen consumption resulting in and preventing the accumulation of reactive oxygen species (Kim *et al*, 2006; Papandreou *et al*, 2006).

In this study, we provide further evidence that PDK-1 is upregulated in hypoxia and that it is under the control of the HIF-1 transcription factor in HNSCC cell lines and many other cancer cell types. We show that PDK-1 contributes substantially to maintaining increased levels of lactate, rather than protecting against free radicals produced under hypoxic conditions from mitochondria. Upregulation of this pathway in head and neck cancer is associated with poor outcome and an aggressive phenotype. Expression of this pathway *in vivo* may help cancer cell survival by maintaining lactate levels and therefore warrants further investigation.

## MATERIALS AND METHODS

### Cell culture

Human colon cell lines CAKI-1, L5174T and SW620 and breast cell line MDA-MB-231 were grown in DMEM. Human HNSCC cell lines TR-138, SCC-4 and SCC-25 were cultured in HAMS F12, DMEM plus 0.4 *μ*g ml^−1^ hydrocortisone and DMEM and HAMS F12 in 1 : 1 plus 0.4 *μ*g ml^−1^ hydrocortisone, respectively. Human renal cell lines RCC4vhl and RCC4ev were grown in α-MEM plus 10 *μ*g ml^−1^ G418. All cell culture medium were supplemented with 10% fetal bovine serum, penicillin (50 IU ml^−1^) and streptomycin sulphate (50 *μ*g ml^−1^). Hypoxic exposure (0.1% O_2_, 5% CO_2_) was performed in a Heto-Holten CellHouse 170 incubator (RS Biotech, Irvine, Scotland). A humidified gas-sorted anoxic incubator-gloved box (InVivo2 400; Ruskin, Leeds, UK) was used for anoxic experiments. The gas was sorted using a Ruskin Microaerophilic gas sorter, resulting in 5% H_2_, 5% CO_2_, and 90% N_2_. Two previously unused palladium catalysts were used to scavenge traces of oxygen. Cell lines were obtained from Cancer Research UK.

### Gene silencing by RNA interference

The target sequences for HIF-1, HIF-2 and scramble control RNAi were selected from the relevant ORF region of the human cDNA sequence according to the manufacturer's recommendations (Cruachem Limited, Glasgow, UK) and submitted to a Basic Local Alignment Search Tool search (National Centre for Biotechnology Information database) to ensure targeting of a single gene. Details of the oligonucleotides (which were purchased from Eurogentec, Southampton, UK) were as follows: HIF-1 antisense, 5′-CUGAUGACCAGCAACUUGAdTdT-3′; HIF-1 sense, 5′-UCAAGUUGCUGGUCAUCAGdTdT-3′; HIF-2 antisense, 5′-CAGCAUCUUUGAUAGCAGUdTdT-3′; HIF-2 sense, 5′ACUGCUAUCAAAGAUGCUGdTdT-3′; control antisense, 5′-ACGACACGCAGGUCGUCAUdTdT-3′; and control sense, 5′-AUGACGACCUGCGUGUCGUdTdT-3′ were synthesised and annealed to form duplexes. The resulting duplexes were transfected at 25 nM into TR-138 cells using oligofectamine reagent (Invitrogen, Paisley, UK) in serum-free Optimem (Invitrogen) for 4 h. The cells were allowed to recover overnight in medium with serum before further treatments.

The target sequence for PDK-1 was selected from the ORF region of the human PDK-1 cDNA sequence using Dharmacon si*DESIGN* Center guidelines and criteria developed by Dharmacon scientists and described by Reynolds *et al* (2004) and submitted to a Basic Local Alignment Search Tool search (National Centre for Biotechnology Information database) to ensure targeting of a single gene. Two oligonucleotides consisting of ribonucleosides with 2′-deoxyribonucleosides (dTdT) at the 3′ ends, 5′-AGUCGCAUUUCAAUUAGAAdTdT-3′ and 5′-UUCUAAUUGAAAUGCGACUdTdT-3′, were synthesised and annealed to form duplexes. The resulting duplexes were transfected at 20 nM as described above.

### Microarray analysis: preparation and labelling of RNA and analysis

HG-U133A Affymetrix GeneChips® were used to profile the HNSCC, SCC-25. The HG-U133A GeneChip consists of 22 283 elements representing 11 870 unique ensemble gene identifiers. SCC-25 cells were transfected with HIF-1, scramble and mock RNAi, and exposed to normoxia and hypoxia for 16 h. Total RNA was extracted using TRI Reagent (Sigma, Poole, UK) followed by reverse transcription using High Capacity cDNA Archive Kit (Applied Biosystems, Warrington, UK). First- and second-strand cDNA synthesis was performed using Superscript ds-cDNA Synthesis Kit (Invitrogen) and 10 *μ*g of total RNA. Clean-up of double-stranded cDNA was carried out using Phase Lock Gels, 2 ml light (Eppendorf, Cambridge, UK), followed by synthesis of labelled cRNA with the BioArray High Yield RNA Transcript Labelling Kit (ENZO, Affymetrix). Purification of cRNA and quantification was done with RNeasy Mini Kit (Qiagen, Crawley, UK), and this was followed by cRNA fragmentation using 30 *μ*g cRNA and fragmentation buffer (200 mM Tris-acetate pH 8.1, 500 mM MKOAc, 150 mM MgOAc). Hybridisation cocktail for human HG-U133A array utilised the Gene Chip Eukaryotic Hybridisation Control Kit (ENZO, Affymetrix), and hybridisation, washing, staining, and scanning of the chip was performed according to the manufacturer's instructions. Data were labelled as MIAME compliant. Raw signal files were background-corrected and normalised using the gcrma modification of the rma normalisation procedure (Irizarry *et al*, 2003) available from the Bioconductor project (www.bioconductor.org) for the R statistical language. Log 2 ratios of hypoxia signal to control signal were generated for each probeset. A list was generated that contained those probesets for which an average absolute fold change of at least 1.5 was observed between normoxic and hypoxic genes, also in addition to a subset of those regulated by HIF-1 and HIF-2. Where a gene was represented by more than one probeset the average signal value was used. This list was used to generate a list of statistically significant (*P*⩽0.01) probesets with the eBayes approach as implemented in the limma package of the BioConductor, which was annotated with regard to gene function and the biological processes present using the gene ontology database (Irizarry *et al*, 2003). An analysis for the statistically significant enrichment of gene ontology terms in this list was carried out to identify functional themes represented.

Visualisation of this gene list was performed using the hierarchical clustering algorithm with euclidean distance and single linkage. The gene list was annotated for gene ontology terms using the GO database and analysis for statistically significant enrichment of terms was performed.

### Immunostaining

Paraffin-embedded tissue samples from 140 consecutive cases of squamous cell head and neck cancer from the ENT department, Radcliffe Infirmary, Oxford, were retrieved and representative tumour areas were mounted on multitissue array slides. Additional tissue sections from apparently normal tongue, buccal mucosa and tonsils were also retrieved for immunohistochemistry. The work was carried out after approval from the Oxford Ethics Committee. All patients were treated with curative intent with primary surgery. The decision regarding postoperative RT was made in a multidisciplinary setting following surgery in conjunction with the histology results. In all 87% (122 of 140) of patients received postoperative RT. Patients and disease characteristics are shown in Table 2.

A modified streptavidin technique was used for immunohistochemistry as previously reported (Koukourakis *et al*, 2006). Sections were deparaffinised and peroxidase was quenched with methanol and H_2_O_2_ 3% for 15 min. Microwaving for antigen retrieval was used (3 × 5 min). The primary anti-POK-1 antibody (KAP-PK112, Bioquote Limited, York, UK) was applied overnight at a concentration of 10 mg ml^−1^. Following washing with TBS, sections were incubated with a secondary antibody (Kwik Kit, Cat. No. 404050, Thermo Shandon, Pittsburgh, PA 15275, USA) for 15 min and washed in TBS. Kwik Streptavidin peroxidase reagent was applied for 15 min and sections were again washed in TBS. The colour was developed by 15 min incubation with DAB solution and sections were weakly counterstained with haematoxylin.

Statistical analysis was performed using the GraphPad Prism 4.0 and the Instat 3.1 package (GraphPad Software Inc., USA). A Fisher's exact test was used for testing relationships between categorical variables (contingency tables) as appropriate. The Kaplan–Meier survival curves were used to assess the impact of various variables in the disease-specific survival of patients. A Cox proportional hazard model was used to assess the effect of assessed parameters on death events. A *P*-value of <0.05 was used for significance.

### Western blotting

Whole cell extracts were made by homogenising cells in lysis buffer (6.2 M urea, 10% glycerol, 5 mM DTT, and 1% SDS plus protease inhibitors). Whole cell extract was separated on 10% SDS-PAGE and transferred to polyvinylidene difluoride membrane. Primary antibodies used were mouse anti-HIF-1α, rabbit anti-HIF-2α (BD Transduction Laboratories, Lexington, KY, USA), goat anti-PDK-1 (Stressgen, Cambridge, UK) and PDH (IgG2a monoclonal antibody against E2/E3 bp and E2 subunits of mitochondrial PDH, Molecular Probes Inc., Eugene, OR, USA), and mouse anti-β-tubulin monoclonal antibody (Sigma, Poole, UK). Immunoreactivity was visualised with horseradish peroxidase-linked goat anti-mouse, anti-rabbit serum or rabbit anti-goat (Dako, Ely, UK) at 1 : 1000 and detected with enhanced chemiluminescence (Amersham, Little Chalfont, UK).

### RNA extraction and reverse transcription

Cells were rinsed with PBS and drained thoroughly. RNA was extracted from the cells using Tri reagent (Sigma, Poole, UK). The quantity and quality of RNA extracted were assessed using NanoDrop ND 1000 Spectrophotometer (NanoDrop Technologies) and the Agilent 2100 Bio-analyzer (Agilent Technologies, West Lothian, UK), respectively. RNA samples were stored at –80°C. cDNA was synthesized by reverse transcribing RNA using the High Capacity cDNA Archive Kit (Applied Biosystems, Warrington, UK) following the manufacturer's instruction.

### Real-time quantitative PCR

Real-time quantitative PCR (qPCR) reactions were performed in triplicate using the Corbett Research Rotor Gene RG-3000 (Sydney, Australia). Each reaction was performed in an individual tube and made up to 25 *μ*l containing 10 *μ*l cDNA, 12.5 *μ*l TaqMan PCR Master Mix (Abgene, Epsom, UK), 0.25 *μ*l probe, 1 *μ*l of forward and reverse primer and 0.2 *μ*l H_2_O. Conditions for the PCR reaction were 2 min at 50°C, 10 min at 95°C and then 40 cycles, each consisting of 15 s at 95°C, and 1 min at 60°C. *β*-Actin was used as a reference gene using primers (Invitrogen) 5′-CCCAGCACAATGAAGATCAA-3′ forward and 5′-CGATCCACACGGAGTACTTG-3′ reverse with probe 63 (Roche, Lewes, UK). Primers against PDK-1 5′-CTGGGTAATGAGGATTTGACTGT-3′ forward and 5′-AAGTCTGTCAATTTTCCTCAAAGG-3′ reverse with probe 10 were used for qPCR. Relative quantitation of gene expression was performed using the method described by Pfaffl (Pfaffl, 2001). In brief, comparisons were made between the number of cycles required for the fluorescence of a sample to reach a predetermined threshold that lay within the exponential phase and above the non-specific background. The relative ratio of gene expression was calculated as follows: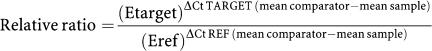 Etarget=reaction efficiency of the gene of interest, Eref=reaction efficiency of the reference gene, Δ*C*_t_=the cycle difference between the comparator and the sample. All calculations are based on the mean value of PCRs performed in triplicate.

### Measurement of mitochondrial dehydrogenase activity in intact cells

Cells were seeded at 2.5–10 × 10^3^ cells per well 100 *μ*l in 96-well plates 24 h prior to experimental treatment in triplicate and incubated in hypoxia or normoxia for 16, 24 and 48 h. Cell viability was measured by measuring metabolic conversion of the dye MTS Cell Titer 96® AQueous One Solution Cell Proliferation Assay (Promega, Southampton, UK). In each well of a 96-well plate, 20 *μ*l of MTS was added and plates were incubated for 2–4 h in a cell culture incubator. MTS assay results were read in a 96-well format plate reader by measuring absorbance at 490 nm.

### Growth curves

TR-138 cells were transfected with siRNA as previously described. After recovery overnight in HAMS F12 with serum, the transfected cells were reseeded in triplicate at 100 000 cells per well of a 6-well plate. The cells were grown for 16, 24, 48 and 72 h in normoxia and hypoxia (0.1% oxygen). The cells were counted using a coulter counter (Beckman, High Wycombe, UK). The media was retained to measure lactate/pyruvate concentrations and stored at −20°C.

### Lactate/pyruvate assay

The lactate and pyruvate concentrations were measured from the media collected from the growth curve experiments, according to the manufacturer's instructions (Instruchemie, Zwet, The Netherlands).

## RESULTS

### Microarray analysis of hypoxia-induced genes

SCC-25 cells were cultured and transfected with the relevant RNAi. All experiments were conducted with HIF-1α, HIF-2α, scramble and mock RNAi transfection, performed simultaneously and exposed to 16 h hypoxia (0.1% pO_2_) or normoxia. RNA was extracted from SCC-25 cells in triplicate. Three separate experiments were performed to minimise the biological variability (Zien *et al*, 2003). The RNA quality was assessed using the Agilent bioanalyser before being converted into labelled cDNA and hybridised to the gene array chip.

The results from the three replicates for each condition were pooled together for the analysis. A total of 392 genes were identified that were upregulated in hypoxia. Of those genes, a number were identified that are regulated by hypoxia and the HIF-1α transcription factor, including BCL2, carbonic anhydrase IX and adrenomedullin. Several genes not previously described at the time as being regulated by hypoxia were also identified, including PDK-1 (Table 1).

### Expression of PDK-1 in different tumour cell lines

**Figure 1:**
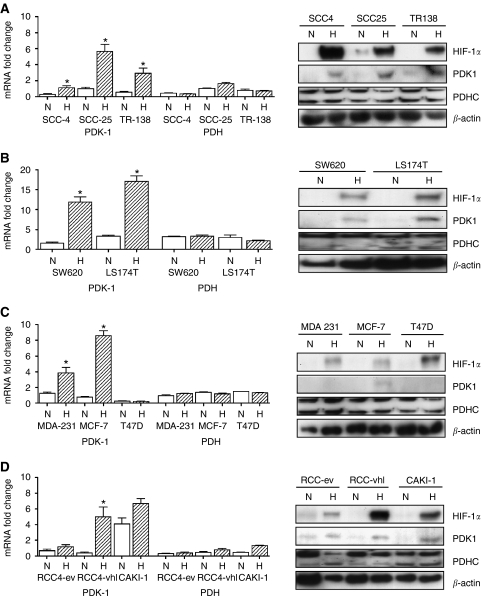
mRNA and protein expression of PDK-1 and PDH in (**A**) head and neck cancer cells, (**B**) colon cancer cells, (**C**) breast cancer cells and (**D**) renal cancer cells. ^*^P<0.05 between hypoxia and normoxia.

To assess whether PDK-1 was specific to HNSCC, the RNA and protein expression of PDK-1 and PDH was quantified in a series of HNSCC cells, colon cancer cells, breast cancer cells, and renal cancer cells (Figure 1).

In three HNSCC cell lines (SCC-4, SCC-25, TR-138) and two colon cancer cell lines (SW620, LS174T), there were similar findings. PDK-1 mRNA and protein expression was increased in hypoxia, but there was no increase in PDH. Of the three breast cancer cell lines, two (MDA-231 and MCF-7) demonstrated an increase in PDK-1 mRNA and protein expression in hypoxia. The third one (T47D) did not demonstrate an increase in mRNA or protein expression of PDK-1 or PDH. In the three renal cancer cell lines tested, RCC4-wt vHL demonstrated an increase in mRNA and protein expression of PDK-1. RCC4-ev, which constitutively express HIF-1α, did not demonstrate any hypoxic upregulation of PDK-1 mRNA. CAKI-1 cells demonstrated an increase in mRNA and protein PDK-1 expression. These findings indicate widespread upregulation of PDK-1 but not PDH in response to hypoxia.

### Regulation of PDK-1 by HIF-1

**Figure 2:**
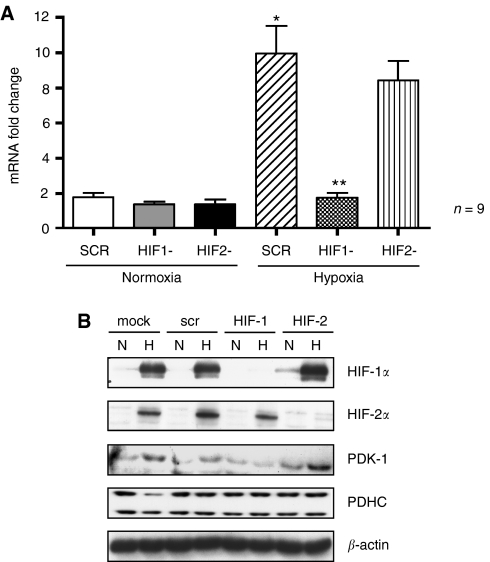
(**A**) mRNA and (**B**) protein expression of PDK-1 in TR-138 cells in normoxia and hypoxia having been treated with scramble (scr), HIF-1α RNAi (HIF1-) and HIF-2α RNAi (HIF2-) siRNA. ^*^ indicates *P*<0.05 between scramble normoxia and hypoxia. ^**^ indicates *P*<0.05 between RNAi-treated cells and scramble-treated cells in hypoxia.

The cell line TR-138 was used for gene silencing experiments. These were conducted with HIF-1α, HIF-2α, scramble and mock transfection, performed simultaneously and exposed to 16 h hypoxia (0.1% pO_2_) or normoxia. Selective gene silencing was confirmed by Q-PCR and western blot analysis (Figure 2A and B). Q-PCR was performed in triplicate and the experiment repeated on three separate occasions such that each data point represents multiple recordings and the s.e. is displayed. All calculations were performed using the mathematical model described by Pfaffl (Pfaffl, 2001).

PDK-1 RNA was significantly (*P*<0.05) upregulated in hypoxia, and HIF-1α RNAi produced a significant (*P*<0.05) reduction in PDK-1 mRNA expression. The mRNA expression of PDK-1 was unaffected in cells treated with HIF-2α RNAi (Figure 2A). PDK-1 protein was also suppressed after HIF-1 RNAi treatment (Figure 2B).

### Effect of PDK-1 knockdown on total cellular dehydrogenase activity

**Figure 3:**
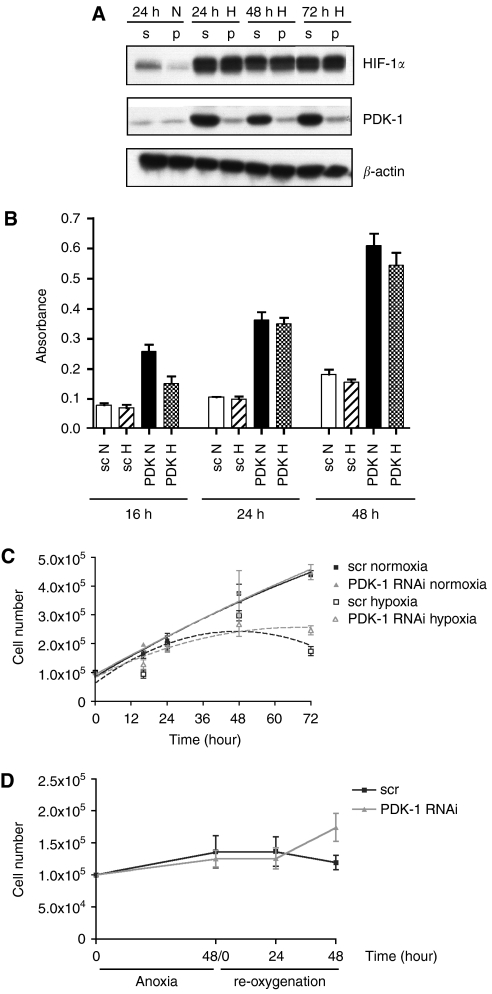
(**A**) RNAi time course for inhibition of PDK-1 (p) expression and scramble siRNA control (s) on PDK-1 expression (**B**) Measurement of PDH activity using CellTiter 96 non-radioactive cell proliferation assay TR-138 cells treated with PDK-1 and scramble (sc) RNAi after 16, 24 and 48 normoxia and hypoxia. (**C**) Cell growth of TR-138 cells treated with PDK-1 RNAi after 16, 24, 48 and 72 h in normoxia and hypoxia, (**D**) Re-oxygenation after 48 h anoxia. Mean and s.e. of three experiments, each performed in triplicate.

The CellTiter 96 non-radioactive cell proliferation assay was used to measure dehydrogenase enzyme activity. Mainly extra mitochondrial dehydrogenases are measured in this assay, and PDH is one of these (Berridge and Tan, 1993; Dunigan *et al*, 1995). Hence, we used this assay as a measure in intact cells of the overall effect of inhibiting PDK-1 using RNAi. PDK-1 gene expression could be silenced using RNAi for over 72 h (Figure 3A). Treatment of TR-138 cells with PDK-1 RNAi resulted in 3–4 fold higher dehydrogenase activity both in normoxia and hypoxia (Figure 3B).

### Effect of PDK-1 knockdown on growth rate in hypoxia

A cell growth assay was used as an initial method of assessing function. Cells were transfected with PDK-1 RNAi before being seeded at a concentration of 100 000 cells in a 6-well plate in triplicate. The cells were then exposed to 16, 24, 48 and 72 h of hypoxia before being counted. Each experiment was repeated three times (Figure 3C). Although there was a general reduction in growth when cells were exposed to hypoxia, there was no significant difference in growth between cells transfected with PDK-1 RNAi and scramble transfection in normoxia or hypoxia.

### Effect of PDK-1 knockdown on growth after reoxygenation after 48 h of anoxia

After 48 h of exposure to anoxia, TR-138 cells treated with PDK-1 and scramble RNAi were reoxygenated for 48 h in an incubator with normal 21% O_2_ (normoxia). During anoxia, there was no significant difference between the growth of scramble or PDK-1 transfected cells. After reoxygenation, there was no significant difference in growth until a minor difference at 48 h reoxygenation (*P*=0.04) (Figure 3D).

### Effect of PDK-1 knockdown on lactate production

**Figure 4:**
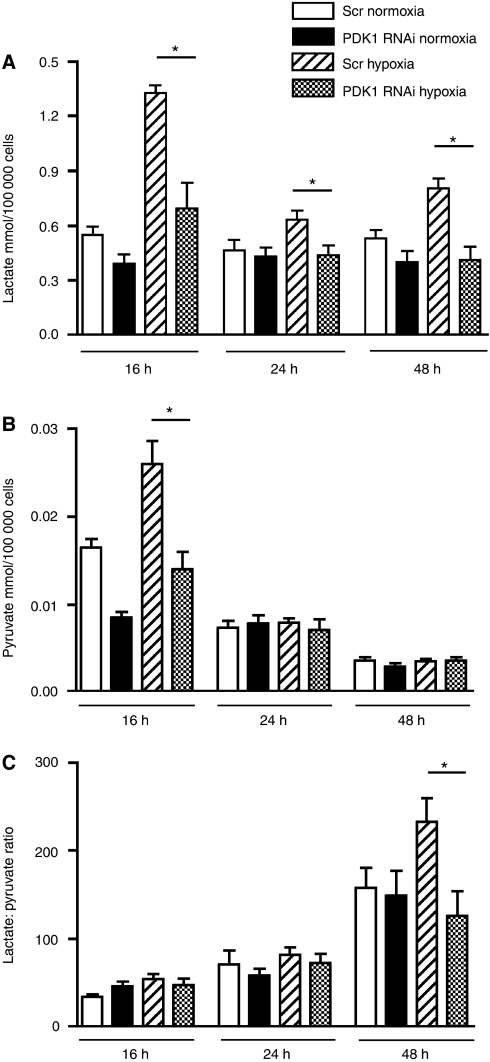
Lactate and Pyruvate assays for metabolites in the conditioned medium of TR-138 cells treated with Scramble (scr) and PDK-1 RNAi. Assayed at 16, 24 and 48 h exposure to 21% oxygen and 0.1% hypoxia for 16 h. (**A**) Lactate and (**B**) Pyruvate (mmol/100 000 cells). (**C**) lactate/pyruvate ratio. ^*^P<0.05. Results of triplicates, representative of three experiments.

The concentration of excreted pyruvate and lactate was measured in the culture media from the cell growth experiments and was corrected for the final cell count number. Each experiment, performed in triplicate, was repeated three times (Figure 4A–C).

After 16 h, there was a significant increase in the excreted lactate concentration between cells exposed to normoxia and hypoxia and treated with a mock or scramble transfection. Cells treated with PDK-1 RNAi and exposed to hypoxia demonstrated a significant (*P*<0.05) reduction in excreted lactate compared to scramble-transfected cells in hypoxia at 16, 24 and 48 h (Figure 4A).

Considering pyruvate production, there was no significant increase after exposure to hypoxia compared to normoxia at any time point. At 16 h, treatment with PDK-1 RNAi compared with scramble transfection significantly reduced the pyruvate levels in cells exposed to hypoxia. Overall, there was a gradual reduction in excreted pyruvate over time in all experimental conditions (Figure 4B).

### Expression of PDK-1 in HNSCC and normal tissues

**Figure 5:**
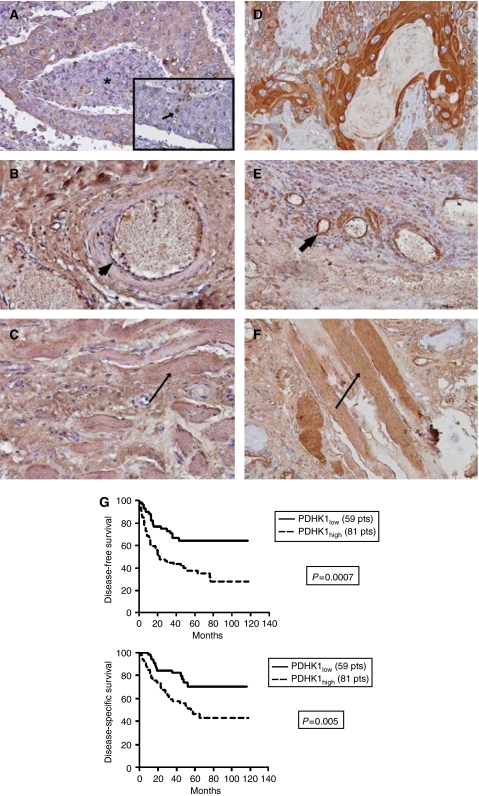
Immunohistochemical expression of PDK-1 (**A**–**C**) and PDH (**D**–**F**). (**A**) PDK-1 expression surrounding necrosis (^*^). Inset nuclear expression (arrow). (**B**) PDK-1 expression in vascular endothelium (large arrow). (**C**) PDK-1 expression in skeletal muscle (small arrow). (**D**) PDH expression in well-differentiated tumour cells. (**E**) PDH expression in vascular endothelium (large arrow). (**F**) PDH expression in skeletal muscle (small arrow). (**G**) Correlation with PDK-1 expression and disease-free survival and disease-specific survival.

To investigate potential clinical relevance, the expression of PDK-1 was assessed in normal and malignant head and neck tissues (Figure 5A–F). The normal tongue or buccal mucosa and submucosa did not express PDK-1. Strong nuclear expression was noted in the laryngeal cartilage and strong cytoplasmic expression in muscular cells. Lymphoid tissue in the tonsils was negative or weakly positive.

PDK-1 was expressed in all cancer cases examined. The staining was mainly cytoplasmic, although nuclear expression (in more than 10% of cancer cells) was noted in 15 of 140 cases. The percentage of cells with strong cytoplasmic expression ranged from 0–100% (median 70%). Using the 70% as a cutoff point, cases were grouped in two categories of low *vs* high PDK-1 reactivity. Cases with nuclear expression were grouped in the high reactivity category regardless of the cytoplasmic expression. Out of 140 cases 81 were considered to bear high PDK-1 reactivity. PDH expression on the same sections had a similar distribution to PDK-1.

PDH was expressed in the cytoplasm of tumour cells. In general well differentiated tumour cells showed stronger immunoreactivity than less-differentiated cells. A similar proportion of cancer cells expressed positive immunoreactivity (71–100%). There was no nuclear expression of PDH in contrast to PDK-1. Considering the expression characteristics in non-tumour cells, PDH was expressed in macrophages, vascular endothelial cells, ductal cells of minor salivary glands, and smooth and striated muscle cells (Figure 5A–F).

Using the Spearman's rank correlation for the percentage of tumour cells with positive immunoreactivity, there was a positive correlation between tumours expressing PDK-1 and PDH (*r*=0.57, *P*=0.03). However, there was no correlation between PDK-1 and CA-9 expression (*r*=0.1, *P*=0.72) or between PDH and CA-9 expression (*r*=0.21, *P*=47).

### Association with histological variables

A marginal association of PDK-1 expression with advanced (T3, 4) local stage was noted (*P*=0.08; Table 2). No association with sex, age, primary location, node involvement, or histological grade was noted.

### Association with prognosis

Out of 81 cases with high PDK-1 expression, 42 (51.8%). relapsed locally or at distant sites *vs* 18 of 59 (30.5%) cases with low PDK-1 expression (*P*=0.01) (Figure 5G). Kaplan–Meier disease-free (a) and disease-specific (b) survival curves showed a significant poorer prognosis of patients with high PDK-1 expression (*P*=0.0007 and *P*=0.005, respectively). The 5-year survival in these cases was 46.4 *vs* 70% of patients with low PDK-1 expression. In multivariate analysis of disease-related death events including T-stage, N-stage and histological grade, PDK-1 expression was the only significant and independent prognostic variable (*P*=0.009, *t*-ratio=2.64).

## DISCUSSION

Hypoxia, through the transcription factor HIF-1, is responsible for the upregulation of many enzymes involved in glycolysis. Here, we describe the identification of PDK-1 as a HIF-1 target protein in HNSCC using a cDNA microarray in SCC-25 cells and show that PDK-1 not only has an effect on pyruvate and lactate metabolism but also that it is associated with a significant poor prognosis in patients with high PDK-1 expression.

Both Q-PCR and western blotting confirmed that PDK-1 was highly upregulated in hypoxia and, furthermore, its expression was significantly reduced following treatment with RNAi against HIF-1α but not HIF-2α, thereby confirming PDK-1 as a HIF-1-dependent target, which is in agreement with the work by Kim *et al* (2006), and Papandreou *et al* (2006). Several cancer cell lines from common types of cancer were analysed and they showed that PDK-1 expression was increased in hypoxia at both the mRNA and protein level in the majority. These findings suggest that PDK-1 is not tissue- or tumour-specific, in spite of its selective expression in a few normal tissues.

To investigate the function of PDK-1, we used gene silencing using RNAi. Because of the problems with assaying PDH enzyme activity in crude extracts (Korotchkina *et al*, 2006), we analysed the function and activity of PDK-1 using the MTS assay. Although this is not a specific assay for PDH, previous studies have shown the utility of analysing the metabolic effects of adding glucose and pyruvate (Segu *et al*, 1998) and also shown that it is mainly the extra mitochondrial enzymes that are measured (Berridge and Tan, 1993). The striking effects in intact cells show the extent of suppression in cancer cells even under basal conditions. Although this assay measures a pool of dehydrogenase activity it is the first demonstration of a change in the activity of the direct target of PDK-1 *in vivo*, as opposed to indirect measures of free radical production or oxygen consumption, which are much smaller.

Although hypoxia reduced the growth of cells, there was no effect of PDK-1 activity on cell growth. These conditions represent chronic hypoxia that can occur in tumours. If this reached the extent of anoxia, we reasoned that on recovery there would be reoxygenation and that a burst of free radicals from mitochondria and PDK-1 may protect from that. Under anoxic conditions, there was no difference in the growth of cells treated with PDK-1 RNAi compared with control-treated cells. Under anoxic conditions, there is no mitochondrial respiration, so a difference in growth rate would not be expected. However, we did observe a small increase in growth rate after 48 h reoxygenation in cells treated with PDK-1 RNAi. Thus, PDK-1 suppression may allow cells to produce more ATP per molecule of glucose utilised, increased fatty acid synthesis and quicker balance of the cells, redox state and NAD cycle.

We did not see the growth inhibitory effects reported by Kim *et al* (2006) or Papandreou *et al* (2006). This may reflect differences in cell types, for example, lymphoid cells studied by Kim *et al* (2006), and the use of hypoxic-activated toxins in Papandreou *et al* (2006).

The excreted lactate and pyruvate were measured during cell growth in normoxia and hypoxia. This revealed an effect of PDK-1 suppression. After 16 h hypoxia, there was a significant increase in lactate and pyruvate concentrations, which were reduced in the cells treated with PDK-1 RNAi.

The level of pyruvate at 16 h was reduced to levels seen in normoxia by silencing PDK-1, indicating that PDK-1 isoform is the principal regulator of the PDH complex in these cells. Importantly, in this study, it was found that with PDK-1 RNAi treatment, after 48 h of exposure to hypoxia, the lactate could be reduced to the level seen in normoxia. This would suggest that the prolonged upregulation of PDK-1 in response to hypoxia and HIF-1α is a key factor in maintaining the elevated lactate and lactate to pyruvate ratio. The additional information in our study of the application of this work to clinical tumour samples showed a major prognostic difference in those tumours with PDK-1 expression. PDK-1 is highly expressed in cardiac, brain, lung, and kidney tissue, but clearly it was differentially expressed in malignant tissues. The expression pattern seen was predominantly cytoplasmic, which is similar to the findings that Koukourakis showed in non-small cell lung cancer (Giatromanolaki *et al*, 2001; Koukourakis *et al*, 2005b). Not previously reported was the finding that PDK-1 demonstrated nuclear expression in a subset of cancers. Although the function of this nuclear fraction is unknown, other glycolytic enzymes have also been reported to show nuclear expression. A high proportion of HNSCC tumours expressed high levels of both PDK-1 and PDH. This is in contrast to a previous study that reported PDH is decreased in epidermal tumours compared to normal epidermis (Eboli and Pasquini, 1994).

The striking and adverse outcome of those tumours with highest PDK-1 expression could be related to a survival benefit on the cancer cells *in vivo*, perhaps indicating that marginal hypoxic/anoxic cells are important for tumour growth. We have previously measured HIF-1α and HIF-2α, as well as carbonic anhydrase 9, the erythropoietin receptor and erythropoietin, and EGF receptor in this series of cases (Winter *et al*, 2005, 2006). None of those markers was as strong a factor in predicting outcome as PDK-1. This may indicate the relative importance of one pathway induced by hypoxia *vs* another one. We recently carried out a gene array analysis of a series of primary head and neck cancers and showed that the hypoxia gene profile differs in every case (Kong *et al*, 2006). Therefore, it is possible that some pathways are biologically more important than others and hence predict outcome better. Another possibility is that it is a robust marker of HIF-1α signalling, not specifically related to its function, the former being shown by several groups to be associated with poor outcome in this and other cancer types. Also, the antigen may be better preserved than HIF and thus more reliable.

We propose a mechanism based on our observation that PDK-1 activity maintains lactate levels in the extracellular medium at about 2-fold higher, most likely by preventing pyruvate metabolism and its entry into the mitochondrial pathway. The high activity of LDHA and monocarboxylate transporters also increased in hypoxia through HIF-1, combined with the inability of cells to convert pyruvate to acetyl CoA by activating PDK-1 results in elevated lactate (Brahimi-Horn *et al*, 2007). Lactate can enhance and maintain HIF activation through inhibition of prolyl hydroxylases (Lu *et al*, 2005). This would have the effect of amplifying the Warburg effect and, indeed, a role of PDK-1 may be to contribute to the effect.

High levels of lactate have been associated with a poor outcome in a number of tumours, including HNSCC (Walenta *et al*, 1997; Brizel *et al*, 2001). However, additionally, LDHA has recently been shown to have a critical role in the energy production of cancer cells through glycolysis, and maintaining this pathway may be a more important aspect of inhibition of PDH (Fantin *et al*, 2006). Recently, Cairns *et al* (2007) have demonstrated that the mitochondrial metabolism of tumour cells is increased by the pharmacologic inhibition of PDK-1. The acute increase in oxygen consumption leads to a corresponding decrease in tumour oxygenation, thereby increasing the effectiveness of some traditional therapies. It will, therefore, be of interest to investigate the relevance of the PDK-1 pathway in *in vivo* models to determine whether inhibitors will be worthwhile to develop clinically and also the relative importance of enhancement of lactate production *vs* suppression of mitochondrial function.

## Figures and Tables

**Table 1:** Hypoxia upregulated genes. Fold change relative to normoxic expression

**Fold change**	**Gene description**
7.2	Enolase 2 (*γ*, neuronal)
6.7	Egl nine homologue 3 (C. elegans)
6.3	Angiopoietin-like 4
6.2	BCL2/adenovirus E18 19 kDa interacting protein 3
6.2	NADH-ubiquitone oxidoreductase MLRQ subunit binding
5.3	Carbonic anhydrase IX
5.3	Lysyl oxidase
4.3	Protein phosphatase 1, regulatory, (inhibitor) subunit 3C
4.2	DNA damage-inducible transcript 4
4.2	Solute carrier family 6 (neurotransmitter transporter, creatine)
3.8	Procollagen-lysine, 2-oxoglutarate 5-dioxygenase 2
3.6	MAX interactor 1
3.4	Cyclin G2
3.2	Jumonji domain containing 1A
3.3	Solute carrier family 2 (facilitated glucose transporter), 1
3.1	Aldolase C
3.1	Phosphoglycerate kinase 1
3.0	Adrenomedulin
2.6	Ephrin A1
2.6	Protein tyrosine phosphatase, receptor type R
2.3	Mucin 1, transmembrane
2.1	Carbonic anhydrase X11
1.8	Pyruvate dehydrogenase kinase 1

**Table 2:** Association of PDK-1 expression with local stage, sex, age, primary location, node involvement or histological grade

		**PDK-1**		
	**No of cases**	**Low**	**High**	***P*-value**
*Gender*				
F	42	20	22	0.45
M	98	39	59	
				
*Age*				
<60	71	33	38	0.30
>60	69	26	43	
				
*Region*				
Hypopharynx	17	6	11	
Larynx	28	15	13	0.55
Oropharynx	46	19	27	
Oral cavity	49	19	30	
				
*T-stage*				
T1	25	15	10	
T2	33	14	19	0.08(^*^)
T3	28	10	18	
T4	54	20	34	
				
*N-stage*				
0	49	20	29	
1	31	12	19	0.81
2	54	25	29	
3	6	2	4	
				
*UICC-stage*				
1	10	4	6	
2	19	9	10	0.35
3	31	15	16	
4a	80	31	49	
				
*Differentiation*				
Good	16	7	9	
Moderate	58	23	35	0.68
Poor	66	29	37	
